# The effectiveness of two doses of recombinant hepatitis E vaccine in response to an outbreak in Bentiu, South Sudan: a case–control and bias indicator study

**DOI:** 10.1016/S1473-3099(24)00657-1

**Published:** 2025-05

**Authors:** Robin C Nesbitt, Vincent Kinya Asilaza, Catia Alvarez, Priscillah Gitahi, Patrick Nkemenang, Jetske Duncker, Melat Haile, Primitive Gakima, Joseph F Wamala, Fredrick Beden Loro, Aybüke Koyuncu, Duol Biem, Manuel Albela, Monica Rull, Etienne Gignoux, John Rumunu, Isabella Eckerle, Iza Ciglenecki, Andrew S Azman

**Affiliations:** a Epicentre, Paris, France; b Médecins Sans Frontières, Juba, South Sudan; c Geneva Centre for Emerging Viral Diseases, Geneva University Hospitals, Geneva, Switzerland; d Department of Medicine, University of Geneva, Geneva, Switzerland; e Médecins Sans Frontières, Geneva, Switzerland; f World Health Organization, Juba, South Sudan; g South Sudan Ministry of Health, Juba, South Sudan; h Department of Epidemiology, Johns Hopkins Bloomberg School of Public Health, Baltimore, MD, USA; i Division of Tropical and Humanitarian Medicine, Geneva University Hospitals, Geneva, Switzerland

## Abstract

**Background:**

Hepatitis E virus (HEV) is a leading cause of acute viral hepatitis, particularly in Asia and Africa, where HEV genotypes 1 and 2 are prevalent. Although a recombinant vaccine, Hecolin, is available, it has not been used to control outbreaks. The licensed three-dose regimen might pose challenges for it to be an impactful outbreak control tool. Our study aimed to estimate the effectiveness of two doses of Hecolin in the context of the first-ever reactive use of the vaccine.

**Methods:**

We conducted a case–control study during an HEV outbreak in the Bentiu internally displaced persons camp, South Sudan. Patients with acute jaundice syndrome (suspected cases) seeking care at the Médecins Sans Frontières hospital were screened for study eligibility. Eligible participants were those that had been eligible for vaccination (ie, living in the camp and aged 16–40 years). Confirmed cases were defined as individuals who tested positive for hepatitis E by RT-PCR or anti-HEV IgM ELISA. Each case was matched to six controls by age, sex, pregnancy status, and residence. Self-reported vaccination status was verified through vaccination cards. The primary analysis was two-dose vaccine effectiveness, which we estimated with a matched case–control design using conditional logistic regression models. In secondary analyses we estimated vaccine effectiveness using a test-negative design and the screening method. We used test-negative cases and their matched controls as a bias indicator analysis to help quantify potential health seeking behaviour biases.

**Findings:**

Between May 10 and Dec 30, 2022, we identified 859 patients with suspected hepatitis E. Of these, 201 met the eligibility criteria and 21 cases had laboratory confirmed hepatitis E. Among the confirmed cases, 10 (48%) were unvaccinated compared with 33 (27%) of 121 matched controls. In the primary analysis we estimated an unadjusted two-dose vaccine effectiveness of 67·8% (95% CI –28·6 to 91·9), and a two-dose vaccine effectiveness of 84·0% (–208·5 to 99·2) after adjustment for potential confounders. The bias indicator analysis suggested that test-negative cases might have been more likely to have been vaccinated than their matched community controls due to different health-care seeking behaviours, potentially meaning underestimation of effectiveness estimates. The test-negative design, which uses facility-matched controls, led to an adjusted two-dose effectiveness of 89·4% (56·4 to 98·0).

**Interpretation:**

Despite the small sample size, our estimates provide evidence of effectiveness of a two-dose regimen against HEV genotype 1 during a protracted outbreak, supporting its use in similar contexts.

**Funding:**

Médecins Sans Frontières.

## Introduction

Hepatitis E virus (HEV) is a common cause of acute viral hepatitis. HEV genotypes 1 and 2, which are transmitted through contaminated food and water, have caused large outbreaks in Asia and Africa.^1^, ^2^ Although the global burden of hepatitis E is difficult to estimate due to poor clinical surveillance and laboratory testing, one widely cited publication suggests that in 2005 there were more than 70 000 deaths attributable to HEV genotypes 1 and 2.^3^

The clinical course of HEV infection is generally self-limited and non-lethal; however, genotype 1 is particularly virulent during pregnancy, presenting substantial risks to maternal and fetal health. Reported case-fatality risks among pregnant women during their second and third trimester are often greater than 25%.^4^ There is no specific antiviral therapy available for the management of hepatitis E caused by these genotypes.

Despite the recognition that safe water and adequate sanitation facilities are the primary preventive strategies for HEV genotype 1 and 2 infections, the communities most vulnerable to outbreaks are typically those furthest from attaining universal water and sanitation access.^5^, ^6^ Fortunately, a three-dose recombinant vaccine, Hecolin (Innovax, Beijing, China), showed excellent efficacy and safety profiles across trials in China, including a large phase 3 trial with more than 100 000 people vaccinated.^7^ This phase 3 clinical trial indicated a 100% protective efficacy against clinically apparent HEV infections (nearly all genotype 4) following the complete three-dose regimen, administered at 0, 1, and 6 months.^7^ Considering these findings, WHO, in 2015, advanced a policy recommendation advocating for the deployment of the hepatitis E vaccine during outbreaks and in settings that present elevated risk, including among pregnant women.^8^

**Research in context**

**Evidence before this study**

We searched PubMed for studies on the effectiveness of the recombinant hepatitis E vaccine Hecolin from database inception to June 20, 2024 using the search terms “Hepatitis E,” “HEV,” “Hecolin,” and “vaccine efficacy”. We included randomised controlled trials, observational studies, and meta-analyses that reported on vaccine efficacy or effectiveness. Our search was not restricted by language.

Previous evidence on vaccine protection comes from a large phase 3 clinical trial conducted in China, which showed that a three-dose regimen of Hecolin had a 100% protective efficacy against clinically apparent hepatitis E virus (HEV) infections over the first 30 months after vaccination with a robust safety profile. The primary study ended 30 months after vaccination but an extended follow-up 10 years after vaccination showed durable protection with three doses. In secondary analyses, no breakthrough infections were identified among the 3792 vaccinees who received only two doses over the first 30 months after vaccination, compared to six infections among two-dose placebo recipients (n=3765). In an extended analysis 10 years post-vaccination, the study identified one breakthrough infection and one additional infection in the two-dose placebo group, yielding a cumulative vaccine efficacy of 89·9%. No other studies evaluating vaccine efficacy or effectiveness have been published.

**Added value of this study**

This study is the first to evaluate the effectiveness of a two-dose regimen of the Hecolin vaccine during an HEV outbreak. Our findings indicate moderate to high two-dose vaccine effectiveness using several study designs and analytical methods; however, the estimates are accompanied with large uncertainty due to the small sample size. This evidence supports the potential for a two-dose regimen to provide substantial protection in outbreak settings, which is crucial given the logistical challenges of administering a three-dose schedule during an outbreak.

**Implications of all the available evidence**

The results of this study, combined with the existing evidence from clinical trials, suggest that a two-dose regimen of the Hecolin vaccine could be an effective and feasible strategy for controlling hepatitis E outbreaks. This strategy has implications for public health policy, particularly in resource-limited settings and populations with high mobility, where completing a three-dose schedule might be impractical. Future research should focus on longer-term follow-up to confirm the duration of protection conferred by two doses and explore the potential for a single-dose regimen in outbreak contexts. Additionally, more studies are needed in diverse epidemiological settings to generalise these findings and optimise hepatitis E outbreak response strategies.

However, a three-dose regimen, with the final dose administered 6 months after the first, might limit the utility of this vaccine in outbreaks. In outbreaks, the third dose might come well after the high-risk period, so protection from the first and second doses are probably key to vaccine impact. Furthermore, outbreaks often occur in settings with high population turnover, making it hard to reach the same people three times. In the phase 3 trial, where the primary endpoint was three-dose efficacy, no breakthrough infections were identified among the 3792 vaccinees who received only two doses over the first 30 months after vaccination, compared with six infections among two-dose placebo recipients (n=3765).^9^ In extended analyses 10 years post-vaccination, the study identified one breakthrough infection and one additional infection in the two-dose placebo group, yielding a cumulative vaccine efficacy of 89·9% (95% CI 43·4–99·7).^9^ Although not a direct marker of protection, anti-HEV IgG antibodies were shown to persist for at least 2 years after vaccination with two doses in a study in Bangladesh.^10^

As of 2022, Hecolin had not been used in an outbreak and no data on clinical protection, with any number of doses, had been published outside of the original phase 3 clinical trial. In 2022, the South Sudan Ministry of Health with the support of Médecins Sans Frontières (MSF) conducted the first hepatitis E vaccination campaign in response to an outbreak.^11^ As part of this campaign, we enhanced surveillance for hepatitis E and conducted a case–control study to measure the short-term effectiveness of a reduced two-dose schedule of Hecolin for preventing medically attended hepatitis E.

## Methods

### Study setting

We conducted a case–control and bias indicator study during an HEV outbreak in the Bentiu internally displaced persons (IDP) camp, South Sudan. The IDP camp originated as a refuge for people fleeing violence at the UN base in Unity State, South Sudan at the end of 2013. In early 2021 the population was estimated to be 101 000. The IDP camp population is mobile with approximately 20% engaging in temporary travel in and out of the camp for employment, education, or other opportunities. MSF runs a secondary hospital with more than 100 beds serving the IDP camp residents and the population of the surrounding towns, and several primary health-care centres operate inside the camp. Despite a robust humanitarian response, access to and use of safe water and sanitation facilities remains a major challenge.

Almost since the inception of the camp, hepatitis E cases have been reported, with notable outbreaks in 2015 (2189 reported cases) and in 2016 (924 reported cases). Low-level hepatitis E transmission continued until August, 2021 when the South Sudan Ministry of Health declared a new outbreak in response to an apparent increase of cases. In response, the Ministry of Health and MSF conducted the first mass reactive vaccination campaign with the Hecolin vaccine in three rounds in March, April, and October, 2022.^12^ The campaign targeted 26 848 residents of Bentiu IDP camp aged 16–40 years, including pregnant women. During the later rounds, the vaccine was offered to anyone within the target group, regardless of whether they received a previous dose or not. Each vaccination round achieved an administrative coverage of over 90%. A coverage survey conducted at the end of the third round estimated that 86% of the vaccine-eligible population had received at least one dose, with 73% and 58% receiving at least two doses and three doses respectively.^12^

Ethical approval was granted for this study from the MSF Ethics Review Board and by the South Sudan Ministry of Health Research Ethics Board as part of the study protocol titled: Effectiveness, safety and feasibility of recombinant hepatitis E vaccine HEV 239 (Hecolin) during an outbreak of hepatitis E in Bentiu, South Sudan. Approval numbers are MSF ERB number 2167 and RERB-MOH number 54/27/09/2022.

### Participants

All individuals with suspected hepatitis E seeking care at the MSF hospital were identified by clinicians in the outpatient, inpatient, and maternity wards and referred to the study team after consultation or were approached after admission. All five primary health-care centres inside the IDP camp referred individuals with suspected hepatitis E to the MSF hospital, which was designated as the main site for care of patients with hepatitis E. Suspected hepatitis E cases were defined as individuals presenting with an acute (recent, new, or sudden) onset of jaundice (yellow coloration of the whites of the eyes or skin, dark urine, or pale clay stools). Study staff explained the study objectives and read the information sheet to each patient with suspected hepatitis E. If the patient with suspected hepatitis E was willing to participate, they provided written informed consent. Individuals younger than 18 years provided written informed assent and their guardians provided written informed consent.

Patients with suspected hepatitis E were eligible for the case–control study if they were residents of the Bentiu IDP camp since the start of vaccination (March 22, 2022), aged 16–40 years during either of the vaccination rounds, and reported that their symptoms started after May 10, 2022.

For each case–control study-eligible case, the study team aimed to recruit six matched controls from the community. Controls were matched on age (±3 years), sex (self-reported by study participants with the options of male, female, or unknown), pregnancy status, and neighbourhood of residence within the Bentiu IDP camp of their matched case. Additional eligibility criteria for controls included camp residence since start of hepatitis E vaccination campaign (March 22, 2022), and no self-report of past suspected hepatitis E or acute jaundice syndrome (recent, new, or sudden onset of jaundice with yellow colouration of the whites of the eyes or skin, dark urine, or pale clay stools). At the end of each day, a list of the cases recruited was compiled with age, sex, pregnancy status, and residence information. The following day, study staff went to the community to recruit the controls. Starting at the household of the case, they proceeded to the next shelter to the right, looking for a matching control. If no one was home, study staff returned within the same day, for up to two visits within 2 days. If no one in the household could be found, or if study staff successfully enrolled a matched control, the next neighbour to the right was chosen until the total number of matched controls were enrolled. Refusals and enrolment were tracked by the study team to help identify protocol violations.

### Procedures

Study staff administered a questionnaire on demographics, recent history of illness, potential risk factors for hepatitis E, and hepatitis E vaccination history to all consenting patients with suspected hepatitis E. If available, a photograph was taken of the hepatitis E vaccination card. To avoid misclassification of hepatitis E vaccination status, study staff showed photos of the unique single-dose vaccine presentation and the green MSF vaccination card and specified the vaccination campaign dates and locations when posing vaccination-related questions. Symptoms and date of onset were self-reported. Before being referred to the laboratory for confirmatory testing and blood sample collection, a follow-up visit 2–4 weeks after the initial consultation was scheduled at the hospital for subsequent testing and vaccination status verification. Participating patients were asked to bring their hepatitis E vaccination card with them to the follow-up visit.

Patients were seen by a study laboratory technician for a series of rapid diagnostic tests (Assure hepatitis E IgM [MP Diagnostics, Eschwege, Germany], SD Bioline hepatitis C [Abbott, Orlando, FL, USA], SD Bioline hepatitis BsAg [Abbott, Orlando, FL, USA], and Paracheck malaria [Orchid Biomedical Systems, Goa, India]) and liver function tests using a Reflotron or SimplexTAS machine (alanine aminotransferase [ALT] and bilirubin) in the hospital laboratory. A venous blood sample (targeting 8 mL) was collected for shipment to the Geneva Centre for Emerging Viral Diseases at the Geneva University Hospitals, Geneva, Switzerland for further testing. Blood samples were centrifuged on site and stored in two separate 2 mL microtubes and frozen at –20°C within 6 h of collection. No rapid diagnostic tests were performed at follow-up, and the same procedure was followed for venous blood samples.

For controls, study staff spoke to the head of each household or another adult and asked if anyone living in the household met the matching criteria. If there was more than one eligible person, one person was selected using a random number generator application (Pretty Random Number Generator for Android, UT, USA) on a tablet. Study staff then spoke directly with the potential participant and their guardian to introduce the study and proceed with the informed consent process. If they were not available, a return visit was scheduled (up to twice before selecting another control household). After confirming that the selected control met the matching and eligibility criteria of the case and providing written consent to participate in the study, study staff administered a questionnaire using a tablet with sections on demographics, vaccination history, recent history of illness and potential risk factors for hepatitis E. If available, a photograph was taken of the hepatitis E vaccination card. No blood samples were collected from controls.

To assign each participant with their effective number of doses, we took the date of each dose and considered a person to be vaccinated by that number of doses 14 days later. For example, if a person received their second dose on January 1 they would be considered vaccinated by one dose until January 15, after which they would be considered to have had two doses. We calculated the effective number of doses on the day of acute jaundice syndrome onset for each case and their matched controls. We conducted sensitivity analyses to understand the impact of this assumed lag to effective protection on our results.

### Laboratory procedures

Plasma samples from all suspected cases were stored in Bentiu hospital laboratory freezers at –20°C for up to 6 months and sent to MSF, Juba, South Sudan, on flights with temperature loggers in cold chain using ultra-frozen ice packs to maintain –20°C for up to 6 h during transport. Samples were then stored in –80°C freezers in Juba until further transport. Samples were sent to Geneva University Hospitals on dry ice with temperature loggers. Upon receipt in Geneva, the samples were stored at –80°C before testing.

RNA was extracted from plasma samples using the NucliSens easyMAG instrument (BioMérieux, Marcy-l'Étoile, France), according to the manufacturer's instructions. The process involves adding lysis buffer to the sample, introducing magnetic silica for nucleic acid binding, followed by a series of wash steps for purification. The elution step includes mixing magnetic silica in the final buffer and using heat for efficient separation of nucleic acid. Final purification entails removing magnetic silica, yielding a pure and concentrated nucleic acid eluate.

We used the ampliCube HEV 2.0 Quant real-time quantitative PCR (rt-qPCR; Mikrogen Diagnotik, Neuried, Germany) system to test for the presence of HEV. This system employs specific primers and probes for hepatitis E virus genotypes 1, 2, 3, and 4, allowing optional quantification by comparing cycle threshold (Ct) values to a standard curve. This system provides a comprehensive approach for the sensitive and specific detection of HEV genomic RNA, including a built-in internal control for quality assurance, optional quantification capabilities, and the ability to simultaneously detect both HEV-specific RNA and the internal control in a single reaction using CFX96 Thermal Cycler (Bio-Rad Laboratories, Hercules, CA, USA). We used a Ct cutoff value for positivity of 42. We also tested for hepatitis A virus with the AltoStar HAV RT-PCR assay (Altona Diagnostics, Hamburg, Germany) following manufacturer's recommendations.

We performed whole-genome sequencing of select samples using the Illumina Viral Surveillance Panel (Illumina, San Diego, CA, USA). Libraries were prepared using the Illumina RNA Prep, with Enrichment (L) Tagmentation reagents and enriched for viral sequences using the Illumina Viral Surveillance Panel (VSP) following the manufacturer's protocols. Total RNA was denatured and converted to double-stranded cDNA, followed by tagmentation, which fragments and tags the DNA with sequencing adapters. Targeted enrichment was achieved by hybridising three-plex library pools with VSP oligonucleotide probes designed to capture sequences from 66 viral pathogens including HEV, followed by amplification and purification of the hybridised fragments. We determined genotype from these sequences using the Hepatitis E Genotyping Tool (version 1.0).

To detect IgM and IgG antibodies we used the Wantai HEV-IgM ELISA (WE-7196) and Wantai HEV-IgG ELISA (WE-7296) kits (Wantai BioPharm, Beijing, China). The specimen's absorbance value (S) is divided by the cutoff value (CO) to obtain the S to CO ratio. If the S to CO ratio is less than 0·9, the result is negative, indicating the absence of HEV infection. A borderline result falls within the range of 0·9 to 1·1 and a positive result is indicated by an S to CO ratio of more than 1·1, pointing to current or past HEV infection.

### Statistical analysis

In our primary analyses, we assessed the protection conferred by two doses of Hecolin against medically attended hepatitis E confirmed through the detection of IgM antibodies (ELISA) or HEV RNA (rt-qPCR). We initially estimated that 39 confirmed cases and 234 matched controls would be needed to estimate two-dose vaccine effectiveness with 90% power and a type I error rate of 0·05 (two-sided). These calculations assumed two-dose vaccine effectiveness of 70% (conservative assumption based on discussions with experts and previous publications^7^, ^13^), a coverage of 70% in the target population (based on conservative expert opinion within MSF), six controls per case, and a correlation in vaccination status of 0·2 between cases and controls. Sample size calculations were done using the epiR package in R (version 2.0.75).

In our prespecified primary analyses, we estimated vaccine effectiveness by contrasting the odds of vaccination between cases and their community-matched controls using conditional logistic regression models (eg, vaccine effectiveness=1–odds ratio) with matched sets treated as strata. In primary analyses of two doses, cases and controls with one dose were not included in the analyses. In our estimates of one-dose effectiveness, individuals with two doses were not included. We estimated 95% CIs from these models using the confint function in R.

In secondary analyses, we used two additional methods to estimate vaccine effectiveness. First, to condition on health seeking behaviour, we used a test-negative design, whereby the odds of vaccination in test-negative and test-positive suspected cases were contrasted using (unconditional) logistic regression models with 95% CIs based on the confint function in R. To reduce misclassification of case status, we classified test-negative cases as those negative by PCR and IgM ELISA, in addition to having a negative rapid diagnostic test. Second, we used the screening method,^14^ where the vaccination coverage in the population is compared to the vaccination coverage among cases. To estimate the proportion of the population vaccinated for the screening method, we used results from a representative vaccine coverage survey conducted just after the second dose campaign (appendix p 7).

We used a directed acyclic graph to determine the set of variables we needed to adjust for in order for the causal odds ratio to be identifiable (appendix p 2).^15^ The minimum adjustment set for the directed acyclic graph was age, time spent in camp, sex, and education level. In the conditional logistic regression models, we did not further adjust for sex because it was perfectly balanced in matched sets. For continuous variables (age and time in camp), we compared models using both linear terms and cubic spline transformations and compared them using Akaike information criteria.

We compared characteristics between cases and matched controls through univariate conditional logistic regression models to account for the non-independence of both groups. When comparing characteristics between cases and unmatched controls we used χ^2^ (categorical where all cell values were ≥5), Fisher's exact tests (categorical where any cell was <5), and Wilcoxon rank sum tests (continuous variables).

To identify potential biases related to differences in those that seek care and those who are in the community, we conducted a bias indicator analysis where we contrasted the odds of vaccination among test-negative cases (ELISA, rapid diagnostic test, and PCR negative) with that of their matched community controls. The expectation, when no systematic biases exist, is that there should be no significant differences in the vaccine coverage between the negative cases and their community matched controls; however, interpreting the magnitude of bias from these estimates is challenging.^16^ As in the primary analysis, we used conditional logistic regression models to estimate the apparent vaccine effectiveness against non-HEV acute jaundice syndrome.

No individual-level missing data were imputed in any analyses. All analyses were conducted in R version 4.3.1.

### Role of the funding source

The funder of the study was involved in the study design, data collection, data analysis, data interpretation, and writing of the report.

## Results

**Figure 1:**
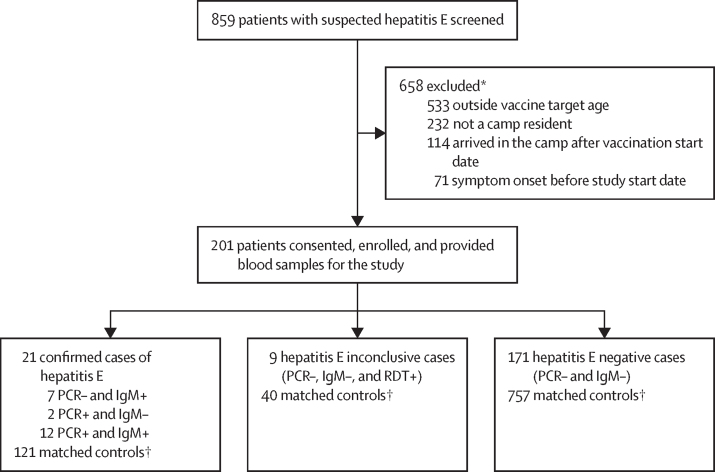
CONSORT flow chart of study enrolment RDT=rapid diagnostic test. *Reasons for exclusion are not mutually exclusive. †We aimed to match six controls per suspected case, but this was not always achieved due to logistical and human resource constraints.

**Figure 2:**
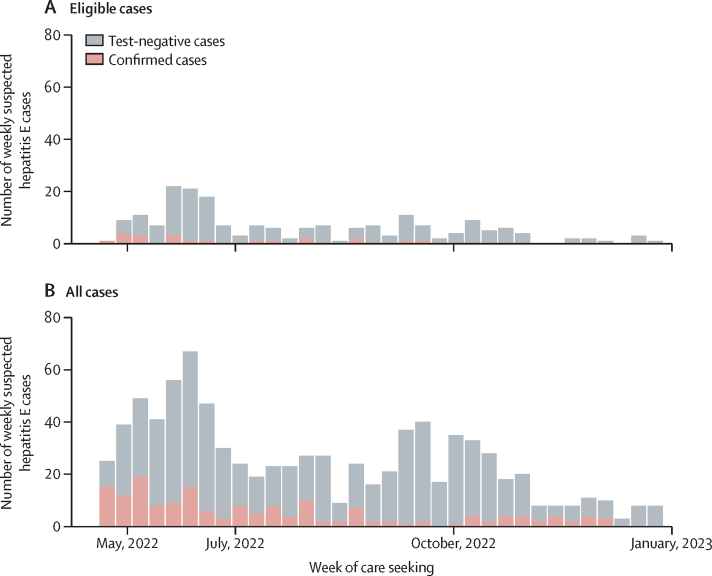
Epidemic curves for suspected cases eligible for study enrolment (A) and all suspected cases regardless of eligibility (B), coloured by hepatitis E case status

From May 10 to Dec 30, 2022, we enrolled 859 patients with suspected hepatitis E including 12 who died while in care at the hospital. Of these, 201 patients met the eligibility criteria for the study and agreed to provide a blood sample (figure 1) and 21 patients had a positive IgM or PCR test, or both (considered confirmed cases). Most patients with suspected hepatitis E were not eligible due to being outside the vaccination target age range (81% of excluded individuals) or did not live inside the camp, or both (figure 1). We enrolled 918 controls matched to the study-eligible cases by age group and residence, 121 of whom were matched to confirmed cases (seven confirmed cases had less than six controls). Confirmed cases were detected from May 13 to Sept 19, 2022, 52 to 181 days after the first round of vaccination (figure 2; appendix p 3). 171 of the enrolled patients with suspected hepatitis E had negative results to all HEV tests (negative cases) and were matched to 757 controls as a bias indicator study (figure 1). We genotyped viruses from 17 PCR positive cases detected in Bentiu during the study period, although none happened to be eligible for this study, and all were genotype 1e.

The 21 confirmed cases had a mean age of 20·9 years (SD 4·9), 14 (67%) were male, and seven (33%) were female (table 1). None of the confirmed cases were pregnant women. Most confirmed cases reported having had a fever (17 [81%]) and dark urine (20 [95%]), although less than half reported having had yellow eyes (10 [48%]; table 2). Symptoms in negative cases were similar to those in confirmed cases, although confirmed cases were 3·8 times more likely to have reported having yellow skin (table 2). Confirmed cases had higher levels of liver-related biomarkers, ALT, and bilirubin compared with the negative cases (table 2; appendix p 4). Of the 21 confirmed cases, 14 (67%) had a positive PCR test (two were IgM negative) and seven (33%) had a positive IgM test only (figure 1; table 2).

**Table 1:** Overview of enrolled cases and controls, including demographics, potential risk factors, and vaccination status

		**Confirmed cases (n=21)**	**Controls (n=121)**	**p value**
Sex		..	..	..*
	Female	7 (33%)	36 (30%)	*..*
	Male	14 (67%)	85 (70%)	*..*
Age, years		20·9 (4·9)	20·7 (3·4)	0·44
Months in the Bentiu internally displaced persons camp		61·3 (21·2–83·0)	86·4 (68·1–94·0)	0·0004
Size of household		8·4 (1·9)	9·0 (3·6)	0·55
Number of children aged <5 years in household		2·0 (1·5)	2·1 (1·6)	0·74
Highest education level achieved		*..*	*..*	<0·0001
	None	15 (71%)	30 (25%)	*..*
	Primary school certificate	5 (24%)	60 (50%)	*..*
	Secondary school or higher	1 (5%)	31 (26%)	*..*
Tap stand drinking water source		21 (100%)	121 (100%)	..*
Defection location		*..*	*..*	<0·0001
	Shared latrine	8 (38%)	116 (96%)	*..*
	Open defecation	13 (62%)	5 (4%)	*..*
Soap in household for handwashing		*..*	*..*	0·57
	Available	9 (43%)	60 (50%)	*..*
	Unavailable	12 (57%)	60 (49%)	*..*
	Unknown	0	1 (1%)	*..*
Household member with acute jaundice syndrome in past 2 months		1 (5%)	7 (6%)	0·57
Shared water source with someone with acute jaundice syndrome		*..*	*..*	0·034
	Yes	4 (19%)	7 (6%)	*..*
	No	17 (81%)	112 (93%)	*..*
	Unknown	0	2 (2%)	*..*
Effective number of vaccine doses†		*..*	*..*	<0·0001
	0	10 (48%)	33 (27%)	*..*
	1	7 (33%)	36 (30%)	*..*
	2	4 (19%)	52 (43%)	*..*
Vaccination card available		6 (55%)	50 (57%)	*..*

Data are n (%), mean (SD), or median (IQR).

* No p value because there was no variation in this variable within matched sets.

† After accounting for lag to protection and the time of case enrolment (for controls).

**Table 2:** Comparison of clinical characteristics, demographic characteristics, and laboratory results between confirmed cases and test-negative suspected cases

		**Confirmed cases (n=21)**	**Negative cases (n=171)**	p value*
**Demographics and risk factors**				
Sex		*..*	*..*	0·05
	Female	7 (33%)	95 (56%)	*..*
	Male	14 (67%)	76 (44%)	*..*
Age, years		20·9 (4·9)	25·5 (6·4)	0·0006
Months in the Bentiu internally displaced persons camp		61·3 (21·2–83·3)	83·6 (53·6–97·0)	0·01
Size of household		8·4 (1·9)	8·2 (2·7)	0·36
Number of children aged <5 years in household		2·0 (1·5)	2·1 (1·3)	0·67
Highest education achieved		*..*	*..*	0·04
	None	15 (71%)	71 (42%)	*..*
	Primary school certificate	5 (24%)	69 (40%)	*..*
	Secondary school or higher	1 (5%)	31 (18%)	*..*
Drinking water source		*..*	*..*	1·00
	Tap stand	21 (100%)	170 (99%)	*..*
	Surface water	0	0	*..*
	Borehole	0	1 (1%)	*..*
Defection location		*..*	*..*	0·041
	Shared latrine	8 (38%)	105 (61%)	*..*
	Open defecation	13 (62%)	66 (39%)	*..*
Soap in household for handwashing		..	..	0·021
	Available	9 (43%)	116 (68%)	*..*
	Unavailable	12 (57%)	54 (32%)	*..*
	Unknown	0	1 (1%)	*..*
Household member with acute jaundice syndrome in past 2 months		*..*	*..*	0·48
	None	20 (95%)	151 (88%)	*..*
	At least one	1 (5 %)	20 (12%)	*..*
Shared water source with someone with acute jaundice syndrome		*..*	*..*	0·26
	Yes	4 (19%)	17 (10%)	*..*
	No	17 (81%)	153 (89%)	*..*
	Unknown	0	1 (1%)	*..*
**Vaccination status**				
Effective doses		*..*	*..*	0·02
	0	10 (48%)	42 (25%)	*..*
	1	7 (33%)	37 (22%)	*..*
	2	4 (19%)	88 (51%)	*..*
	3	0	4 (2%)	*..*
Vaccination card available		6 (55%)	70 (54%)	1·00
**Jaundice and care seeking**				
Who noticed jaundice		*..*	*..*	0·56
	Self	7 (33%)	68 (40%)	*..*
	Someone else	14 (67%)	102 (60%)	*..*
	Unknown	0	1 (1%)	*..*
Sought care before hospitalisation		11 (52%)	114 (67%)	0·19
Past hepatitis diagnosis		0	7 (4%)	1·00
Past acute jaundice syndrome		0	4 (2%)	1·00
**Symptoms**				
Fatigue		2 (10%)	18 (11%)	1·00
Loss of appetite		4 (19%)	55 (32%)	0·22
Nausea		3 (14%)	19 (11%)	0·71
Vomiting		6 (29%)	31 (18%)	0·25
Fever		17 (81%)	137 (80%)	1·00
Diarrhoea		5 (24%)	41 (24%)	0·99
Epigastric pain		4 (19%)	45 (26%)	0·47
Itch		0	3 (2%)	1·00
Joint pains		11 (52%)	84 (49%)	0·78
Bleeding		0	2 (1%)	1·00
Other symptoms		13 (62%)	107 (63%)	0·95
Yellow eyes		10 (48%)	109 (64%)	0·15
Yellow skin		7 (33%)	15 (9%)	0·0040
Dark urine		20 (95%)	147 (86%)	0·32
Pale stools		0	1 (1%)	1·00
**Laboratory results**				
Bilirubin, mg/dL		4·0 (1·1–7·8)	0·5 (0·5–0·7)	<0·0001
	Unknown	11 (52%)	39 (23%)	*..*
Alanine aminotransferase result, IU/L		23·3 (15·6–661·0)	16·0 (12·3–23·9)	0·017
	Unknown	8 (38%)	50 (29%)	*..*
HEV PCR test result		..	*..*	<0·0001
	Positive	14 (67%)	0	*..*
	Negative	7 (33%)	171 (100%)	*..*
ELISA anti-HEV IgM test result		*..*	*..*	<0·0001
	Positive	19 (90%)	0	*..*
	Negative	2 (10%)	171 (100%)	*..*
ELISA anti-HEV IgG test result				0·56
	Positive	20 (95%)	165 (96%)	*..*
	Negative	1 (5%)	5 (3%)	*..*
	Indeterminate	0	1 (1%)	*..*
Malaria rapid test result		..	..	0·38
	Positive	0	15 (9%)	*..*
	Negative	21 (100%)	156 (91%)	*..*
Hepatitis A virus PCR test result		*..*	*..*	1·00
	Positive	0	0	*..*
	Negative	21 (100%)	171 (100%)	*..*
HBsAg rapid test result		*..*	*..*	0·79
	Positive	4 (19%)	43 (25%)	*..*
	Negative	15 (71%)	122 (71%)	*..*
	Unknown	2 (10%)	6 (4%)	*..*
Hepatitis C virus rapid test result		*..*	*..*	1·00
	Positive	0	2 (1%)	*..*
	Negative	21 (100%)	169 (99%)	*..*

Data are n (%), mean (SD), or median (IQR). This table excludes the nine people who had inconclusive outcomes and four negative cases who had three doses of vaccine (excluded from analyses of one and two doses). HEV=hepatitis E virus.

* Pearson's χ^2^ test, Wilcoxon rank sum test, or Fisher's exact test.

Compared with their community controls, cases had resided in the camp for a shorter time, were less likely to be literate, more likely to share a water source with someone with acute jaundice syndrome, and more likely to have reported open defecation (table 1). All cases and controls reported using water from a centralised water system in the camp as their primary drinking water source. Cases were more likely to be unvaccinated (10 [48%] of 21) compared with their matched controls (33 [27%] of 121). Four (19%) cases had two vaccine doses compared with 52 (43%) controls. Just over half of cases (six [55%] of 11) and controls (50 [57%] of 88) who reported to have been vaccinated were able to provide a vaccination card.

None of the vaccine breakthrough cases were admitted to the hospital compared with two of the ten unvaccinated cases (appendix p 8). Median ALT levels in vaccinated cases (31·9 IU/L [IQR 15·5–661·0]) were similar to those in unvaccinated cases (21·7 IU/L [17·4–1140·0). Median bilirubin levels in unvaccinated cases (6·8 mg/dL [1·1–12·0]) were more than three times that of vaccinated cases (2·2 mg/dL [1·0–4·0], although this difference was not significant (p=0·34). Vaccinated cases appeared to have similar viral loads and Ct values as unvaccinated cases (p=0·76; appendix p 8).

We conducted several analyses aimed at estimating vaccine effectiveness (table 3). In our primary analyses, with community-matched controls, we estimated an unadjusted two-dose effectiveness of 67·8% (95% CI –28·6 to 91·9), and a two-dose effectiveness of 84·0% (–208·5 to 99·2) after adjusting for potential confounders (age, education level, and time in camp). In our bias indicator analysis, we found those seeking care for non-hepatitis E acute jaundice were more likely to be vaccinated than their matched controls in the community (eg, 30·3% unvaccinated in controls and 24·6% unvaccinated in test-negative cases; appendix pp 9–10), suggesting the potential that our effectiveness estimates were biased towards the null due to health seeking practices.

**Table 3:** Overview of vaccine effectiveness results for different design and analytical methods

	Number of cases (number effective)*	**Number of controls (number effective)**†	**Number of vaccinated cases (%)**	**Number of vaccinated controls (%)**	**Unadjusted vaccine effectiveness (95% CI)**	**Adjusted vaccine effectiveness (95% CI)**‡
**Community-matched case–control study**§						
Two doses	14 (11)	52 (47)	4 (29%)	28 (54%)	67·8% (−28·6 to 91·9)	84·0% (−208·5 to 99·2)
At least one dose	21 (17)	121 (97)	11 (52%)	88 (73%)	60·1% (−9·9 to 85·5)	63·3% (−43·8 to 90·6)
One dose	17 (14)	51 (49)	7 (41%)	27 (53%)	56·7% (−46·3 to 87·2)	63·6% (−87·9 to 93·0)
**Test-negative design, facility controls**¶						
Two doses	14 (14)	130 (130)	4 (29%)	88 (68%)	80*·9*% (39·3 to 95·0)	89·4% (56·4 to 98·0)
At least one dose	21 (21)	171 (171)	11 (52%)	125 (75%)	64*·2*% (8·4 to 85·9)	67·5% (1·5 to 89·6)
One dose	17 (17)	79 (79)	7 (41%)	37 (47%)	20*·5*% (−128·0 to 73·6)	31·9% (−136·2 to 81·8)

* Effective controls are the number of controls that contribute to likelihood function in each model estimating vaccine effectiveness. In the case of two-dose vaccine effectiveness, only individuals with either zero or two doses of vaccine contribute information to the likelihood and individuals with one dose are effectively removed from the model. In the case of one-dose vaccine effectiveness, only individuals with one or zero doses contribute to the likelihood.

† Although we aimed to enrol six controls per case, this was not always feasible, and some cases have slightly more or less than six controls. Three of the cases had more than six matched controls and seven had less than six.

‡ Adjusted for age, sex, education, and months living in camp in test-negative design; and adjusted for age, education, and months living in camp in community-matched analysis.

§ Primary analysis using a matched case–control design and conditional logistic regression.

¶ Test-negative design using facility controls and unconditional logistic regression.

We then estimated vaccine effectiveness using a test-negative design, contrasting the odds of vaccination in test-positive and test-negative cases reporting for care at the MSF hospital, which might not be as affected by health seeking behaviour biases. In these analyses we estimate an unadjusted two-dose vaccine effectiveness of 80·9% (95% CI 39·3 to 95·0) and after adjustment for potential confounders, 89·4% (56·4 to 98·0). In a secondary analysis using the screening method, which contrasts the expected vaccine coverage in the community, as measured by a vaccine coverage survey (appendix p 7), and the coverage in cases, we estimate a vaccine effectiveness of 72·2% (17·0 to 92·4) for two doses and 52·4% (–38·9 to 75·6) for at least one dose. We found that estimates of two-dose vaccine effectiveness were qualitatively similar using varying assumptions about the time to effective protection from each dose (0–8 weeks; appendix pp 5–6).

Using our primary analysis approach, we estimate the unadjusted one-dose effectiveness to be 56·7% (95% CI –46·3 to 87·2) and an adjusted effectiveness of 63·6% (–87·9 to 93·0). When using the test-negative design, unadjusted effectiveness unexpectedly drops to 20·5% (–128·0 to 73·6) and adjusted effectiveness drops to 31·9 (–136·2 to 81·8), although with extremely wide confidence intervals. However, in sensitivity analyses when considering longer times to effective protection, estimates of one-dose protection increase with longer assumed lags, reaching more than 50% after 4 weeks, similar to estimates from the matched analyses (appendix pp 5–6).

We conducted several analyses to explore the impact of using different transformations of the continuous predictors in the adjusted regression models and alternative confirmed HEV case definitions. Using restricted cubic splines for the continuous variables (age and time spent in camp) led to similar estimates of vaccine effectiveness across doses and analytic methods (appendix p 10). Alternative case definitions led to qualitatively similar results for two-dose vaccine effectiveness (appendix p 11).

## Discussion

Our motivation for this study was to better understand the potential for Hecolin as an outbreak control tool. Vaccination campaigns with the current formulation of Hecolin come with heavy logistical and resource needs,^11^ especially in areas where cold-chain capacity is limited. Maintaining or resetting up temporary infrastructure to support the third round of vaccination, 5 months after the second round, could make vaccination in outbreaks resource prohibitive. Furthermore, if the third dose is indeed needed for protection, vaccine impact might be limited in outbreaks, because individuals might only start to benefit after the high-risk period is over. In 2024, WHO updated their 2015 position on hepatitis E vaccines, and suggested that two-dose regimens should be considered in outbreaks, based on the immunogenicity studies and limited clinical trial data.^17^ Our study provides further support for this recommendation. A two-dose approach might open the door to more reactive use of the vaccine, perhaps without compromising short-term protection, which is most needed in acute outbreaks.

Although the sample size in our study was lower than we had planned for, we explored various approaches to estimating effectiveness and while some estimates come with large uncertainty, they all consistently point towards substantial protection from this regimen. We used prespecified conditional logistical regression models for our main analyses. However, there has been much debate on both the value and consequences of both matched designs and matched analyses in the epidemiological literature over the past decades.^18^ In particular, matched analyses can lead to reductions in precision due to loss of effective sample size when case–control sets have concordant exposures, as is seen also in our analyses (table 3). The bias indicator analyses led to negative point estimates, highlighting the potential for underestimation of effectiveness when using community-matched controls, although confidence intervals were extremely wide. When we estimated vaccine effectiveness with an unmatched design, the point estimates increased, and confidence intervals were far narrower, as expected.

A secondary aim of our study was to understand the protection conferred by one dose of the vaccine. Studying one-dose protection can be challenging because there is only a 1-month period between the first and second dose, leaving only a few people having received a single dose (16% based on our coverage survey). Estimates from our primary analysis point towards similar levels of protection from one and two doses, although the bias indicator analysis point estimates suggest that they could be underestimates (bias towards the null). However, unlike for two doses, the point estimate from the test-negative design did not increase compared with analysis with matched community controls; the reasons for which are unclear. In our main analyses we assumed that protection from each dose started 2 weeks after being vaccinated; however, the lag to effective protection after the first dose might be longer than after the second due to previous priming of the immune system. Given the long incubation period of hepatitis E (ie, genotype 1 infections with a median of 30 days^19^), it is possible that cases were vaccinated after having been infected and that we misclassified the effective protection onset time for both cases and controls. We conducted sensitivity analyses to explore the potential impact of this by shifting the lag between vaccination and effective protection and found that with lags greater than 1 month, estimates of one-dose effectiveness from both the test-negative and matched case control designs were consistent and above 50% (appendix pp 5–6), suggesting the possibility one-dose vaccine effectiveness might have been underestimated. More work is needed to better understand one-dose protection and the effect of post-exposure vaccination against hepatitis E.

Although not the primary focus of this study, we found important differences between cases and both community and health-facility controls pointing towards the potential roles of education, sanitation and hygiene behaviours, and mobility shaping hepatitis E risk. Cases were less likely to have had a formal education than their controls. Cases were far more likely to report open defecation, share a water source with someone with acute jaundice syndrome (but not living with someone with acute jaundice syndrome), and were less likely to have soap on site for handwashing than their controls. If causal, the mechanism for the relationship between open defecation and hepatitis E is unclear; however, it might simply be an indicator of participants' general hygiene behaviours. Although most participants had been living in Bentiu for multiple years, cases, on average, reported having lived in the camp for less time than controls, which could be related to differences in historical exposure or infection risk. Notably, we had almost no variation in water sources within this study, so we were unable to explore how water source influenced hepatitis E risk.

This observational study comes with several limitations. First, despite our attempt to enrol all eligible cases, our final sample size was small, with only 21 confirmed cases. In our primary analysis of two-dose effectiveness, the number of cases were further reduced, because those who had received one dose of vaccine (cases and controls) were censored as were vaccine concordant case–control sets. We chose to use a matched design to help ensure that we had balance in the distributions of confounders, such as age and sex, but matching can induce biases, which often require further adjustment in analyses.^18^, ^20^, ^21^ This small sample size led to wide confidence intervals and limited our ability to test more complex regression models to control for confounding and stratified analyses (eg, by age, severity of disease, and sex). We attempted to control for confounding guided by the use of a simple directed acyclic graph but we cannot rule out confounding by unmeasured variables or misspecification of our directed acyclic graph. Although some of our estimates come with large statistical uncertainty, the multiple analyses, using different analytical approaches and different outcome definitions all point towards similar qualitative conclusions about protection from two doses. Because there was no central database or register of vaccinees, we relied on self-reporting, usually with vaccination card proof, to ascertain vaccination status. While we cannot rule out misclassification of vaccination status, the fact that this study was conducted within the first 8 months of the campaigns and that these campaigns targeted a unique population of people aged 16 years and older, as opposed to children, provides some reassurance that people would remember being vaccinated. Our primary analyses used a permissive case definition allowing for acute jaundice syndrome cases with either a positive IgM or PCR test to be considered cases. Although there are limited data on IgM responses post-vaccination, analyses from China and the USA suggest that some vaccinated cases might have an IgM response for several months after vaccination.^22^, ^23^ Given this risk of misclassification, we explored alternative case definitions including one based on PCR positivity only and found similar results (appendix p 11). Our study was conducted in the context of a protracted outbreak, where cases had been reported, almost continuously, since 2015. Although we did not test controls, or the general population, for anti-HEV IgG antibodies indicative of past exposure, we found that 90% of unvaccinated HEV negative cases were positive for anti-HEV IgG antibodies suggesting high levels of previous exposure. This immunological background might have increased the protection from one dose and two doses of the vaccine, compared with what might be expected in a more immunologically naive population. Future work looking at the avidity of IgG antibodies might help further characterise the proportion of the population who was previously infected.

One of the ultimate aims of reactive vaccination is to stop or slow outbreaks. While the outbreak in Bentiu continued after the vaccination campaigns, there were almost no cases within the vaccine target population during the surveillance period (appendix p 3). While more in-depth work is needed to quantify the impact this vaccination campaign may have had, it seems evident that the target population was too limited to have a major influence. The campaign was focused only on camp residents due to the small number of doses available. However, there is substantial mixing of populations in and outside the camp and a broader vaccine focus might have led to more evident reductions in the incidence of hepatitis E. Hecolin is currently licensed for individuals aged 16 years and older. In screening for eligibility for our study, 70·6% of PCR confirmed cases were younger than 16 years and 26·7% were aged five years or younger. While the age distribution of infections is certain to vary across epidemiological settings, future work to expand the age range for vaccination might be key to realising large population-level impacts. Finally, while not evident in our study population, if two doses do not provide sufficient protection in some populations, accelerated three-dose schedules might be one way to reduce delays to effective immunity.^24^

In conclusion, our results suggest high protection of a two-dose schedule of Hecolin when used in response to an outbreak of hepatitis E genotype 1. Although more data on vaccine protection are needed from different epidemiological settings and for longer durations, these results lend support to WHO's recently updated position on hepatitis E vaccination. Considering the WHO position paper and the small but reassuring body of evidence on two-dose protection, including that presented here, countries affected by hepatitis E outbreaks should consider the use of vaccines in the challenging battle against this disease.

### Contributors

### Data sharing

The minimal dataset underlying the findings of this paper are available on request, in accordance with the legal framework set forth by MSF data sharing policy. MSF is committed to sharing and disseminating health data from its programmes and research in an open, timely, and transparent manner to promote health for populations while respecting ethical and legal obligations towards patients, research participants, and their communities. The MSF data sharing policy ensures that data will be available upon request to interested researchers while addressing all security, legal, and ethical concerns. Readers can contact the MSF generic address data.sharing@msf.org or the Epicentre generic address epimail@epicentre.msf.org to request the data that can be shared with researchers subject to the establishment of a data sharing agreement to provide the legal framework for data sharing.

## Declaration of interests

MSF provided support in the form of salaries for ASA, VKA, PGi, PN, JD, MH, PGa, MA, MR, and IC and indirectly provided salary support for Epicentre employees RCN and EG. ASA serves as a member of the Gavi, the Vaccine Alliance Independent Review Committee. All other authors declare no competing interests.
