# Supplementary material for: The effectiveness of two doses of recombinant hepatitis E vaccine in response to an outbreak in Bentiu, South Sudan: a case–control and bias indicator study
Source: Lancet Infect Dis. 2025 May;25(5):537–47. doi: 10.1016/S1473-3099(24)00657-1 (PMC12018298; doi:10.1016/S1473-3099(24)00657-1)
Supplement: Supplementary appendix [file mmc1.pdf]

THE LANCET
Infectious Diseases

Supplementary appendix

This appendix formed part of the original submission and has been peer reviewed. We post it as supplied by the authors.

Supplement to: Nesbitt RC, Kinya Asilaza V, Alvarez C, et al. The effectiveness of two doses of recombinant hepatitis E vaccine in response to an outbreak in Bentiu, South Sudan: a case–control and bias indicator study. *Lancet Infect Dis* 2025; published online Jan 8. https://doi.org/10.1016/S1473-3099(24)00657-1.

The Effectiveness of Two Doses of Recombinant Hepatitis E Vaccine in Response to an Outbreak in Bentiu, South Sudan: A Case-Control and Bias Indicator Study

Supplemental Appendix

Robin C. Nesbitt, Vincent Kinya Asilaza, Catia Alvarez, Priscillah Gitahi, Patrick Nkemenang, Jetske Duncker, Melat Haile, Primitive Gakima, Joseph F. Wamala, Fredrick Beden Loro, Aybüke Koyuncu, Duol Biem, Manuel Albela, Monica Rull, Etienne Gignoux, John Rumunu, Prof Isabella Eckerle, Iza Ciglenecki, Andrew S. Azman

Table of Contents

Figure S1. **Directed Acyclic Graph developed to help decide on key adjust variables for the study**.......... 2
Figure S2. **Overview of medically attended confirmed hepatitis E cases (IgM or RT-PCR positive)**. 3
Figure S3. **ALT distribution of HEV negative, HEV positive and Indeterminate cases in study by sex**. 4
Figure S4. **Sensitivity analyses on assumed lag to protection by each vaccine dose.**.......... 5
Figure S5. **Estimates (unadjusted) of one-dose protection over different assumed lag-times from test-negative design**.......... 6

Table S1 **Vaccine coverage estimates from after the second vaccination round.**.......... 7
Table S2. ***Comparison of characteristics and lab results from vaccine breakthrough and non-breakthrough confirmed cases.*** 8
Table S3. **Overview of enrolled test negative cases and their matched controls (bias-indicator study), including demographics, potential risk factors and vaccination status.** 9
Table S4. **Estimates from bias indicator study using community controls and conditional logistic regression models** 10
Table S5. **Estimates of vaccine effectiveness (VE), lower and upper 95% confidence intervals, and AIC for models with restricted cubic splines for continuous variables**. 10
Table S6. **Effectiveness estimates with alternative case definition where a case is any suspected case with ALT elevated ≥2.5 normal limit with at least one positive HEV test (ELISA IgM, RDT IgM, PCR or 4-fold IgG rise)**.......... 11

Supplemental Figures

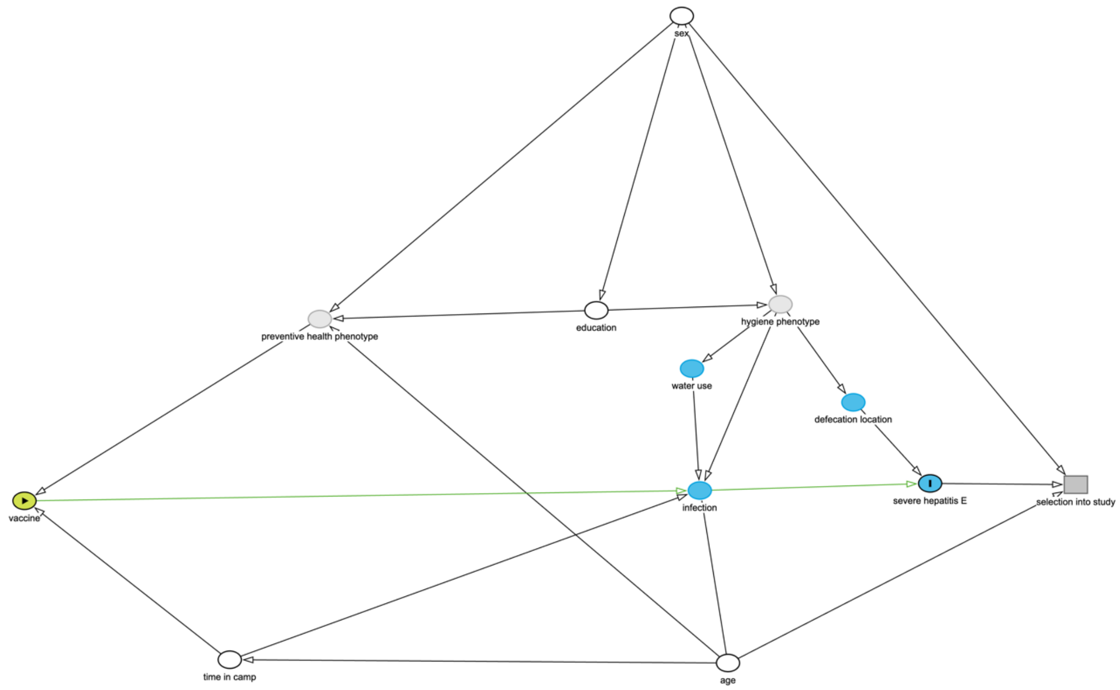

Figure S1. **Directed Acyclic Graph developed to help decide on key adjust variables for the study**. DAG was constructed and analyzed with dagitty.net. Path of causal odds ratio shown in green. White circles represent variables that are adjusted for, blue are those unadjusted for, grey are latent variables and green is the exposure of interest.

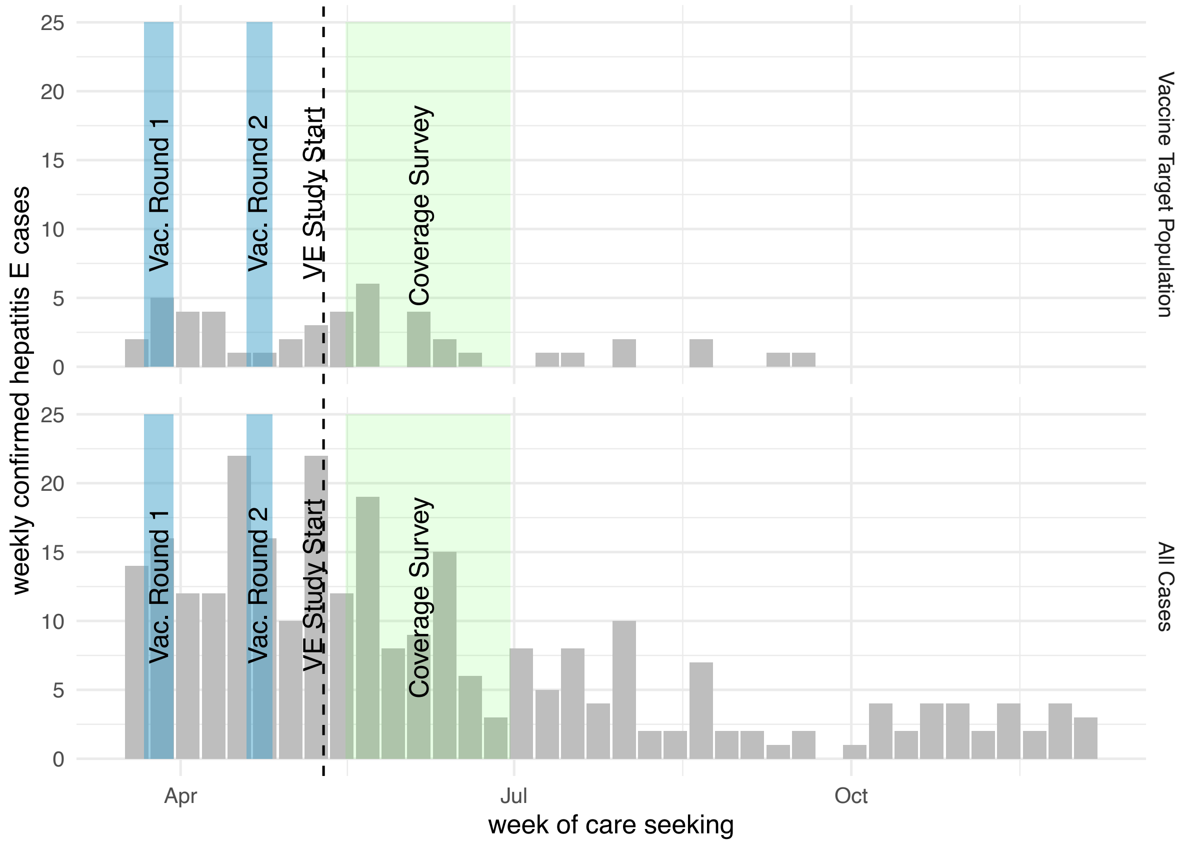

Figure S2. **Overview of medically attended confirmed hepatitis E cases (IgM or RT-PCR positive)**. Top panel illustrates cases who would have been eligible for vaccination (thus meeting most criteria for entry into the case control study) and the bottom panel represents all confirmed cases. VE: vaccine effectiveness.

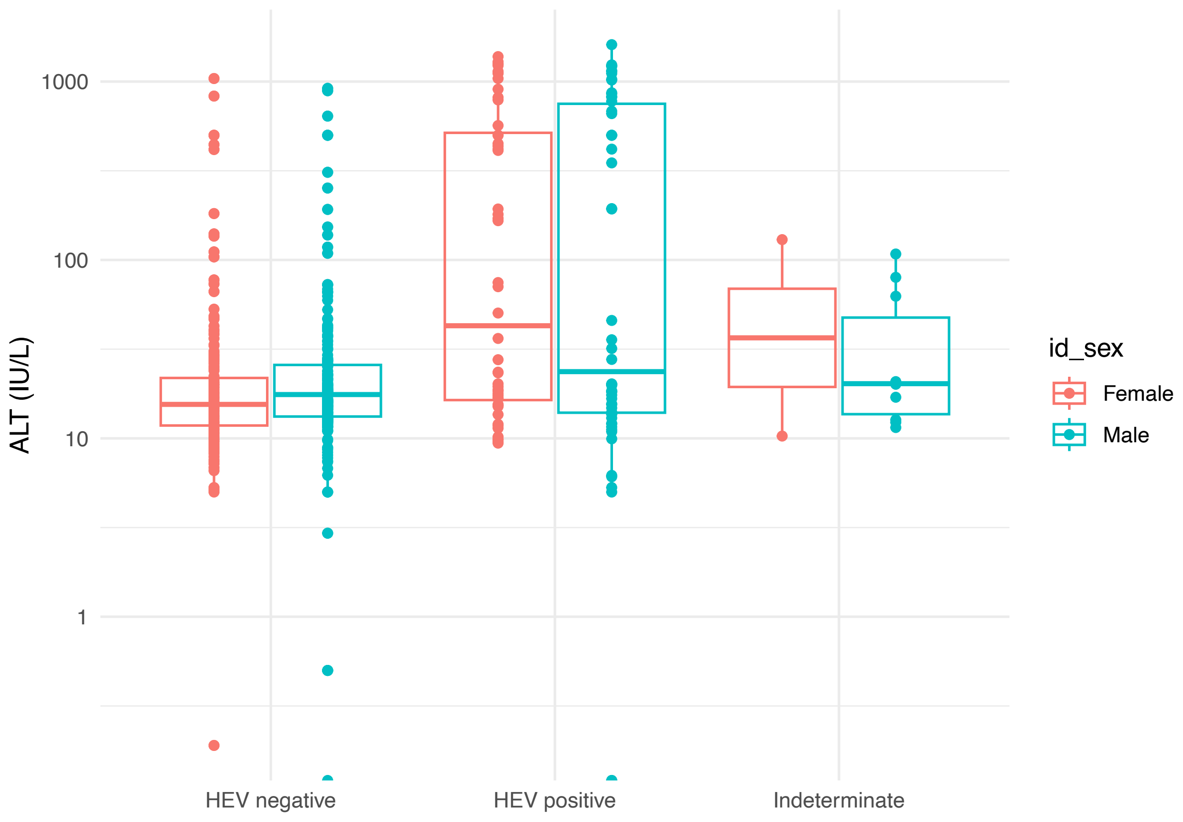

Figure S3. **ALT distribution of HEV negative, HEV positive and indeterminate cases in study by sex**. Box plots show the median and interquartile *range of the distribution of ALT for each test result and sex class.*

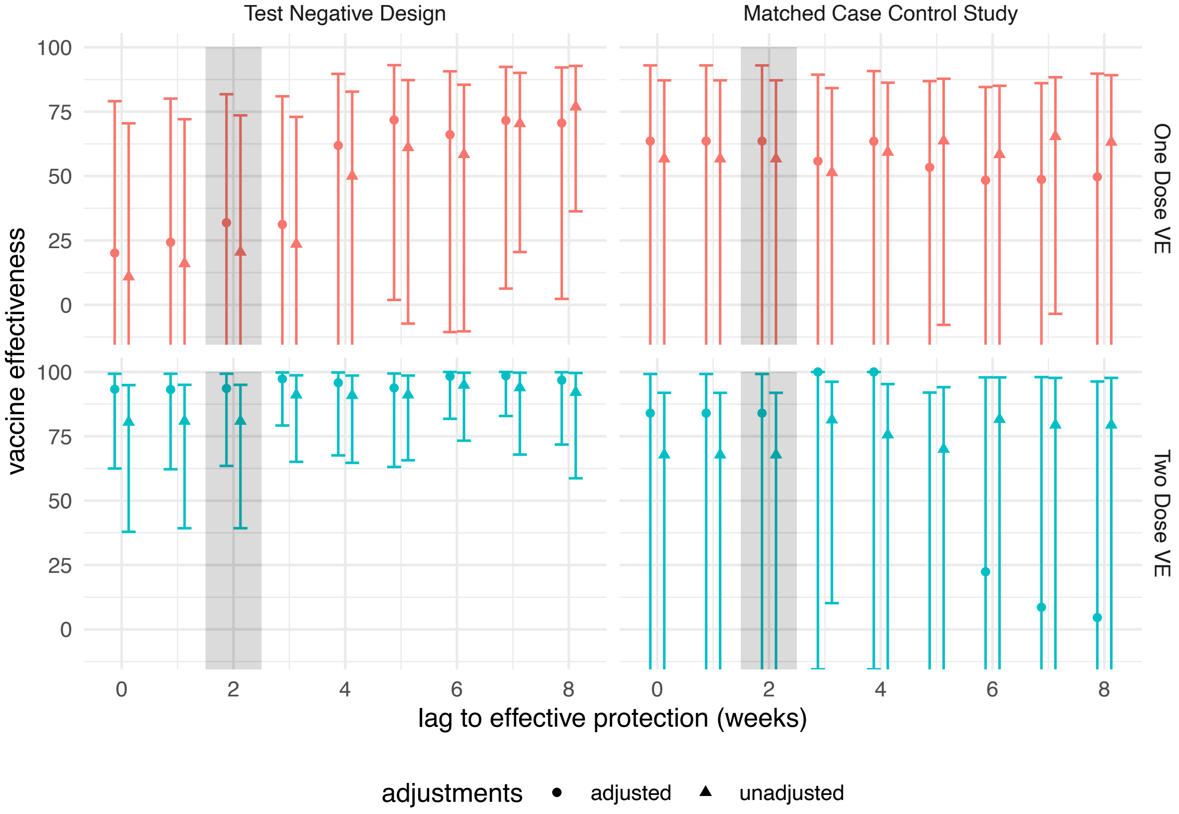

Figure S4. **Sensitivity analyses on assumed lag to protection by each vaccine dose.** Points represent point estimates with round shapes illustrating unadjusted models and triangles with adjusted models. 95% confidence intervals for each point estimate are shown though the lower limit of the y-axis is truncated. Vertical dashed line represents the assumed lag used in the main analyses (specified a priori). VE: vaccine effectiveness.

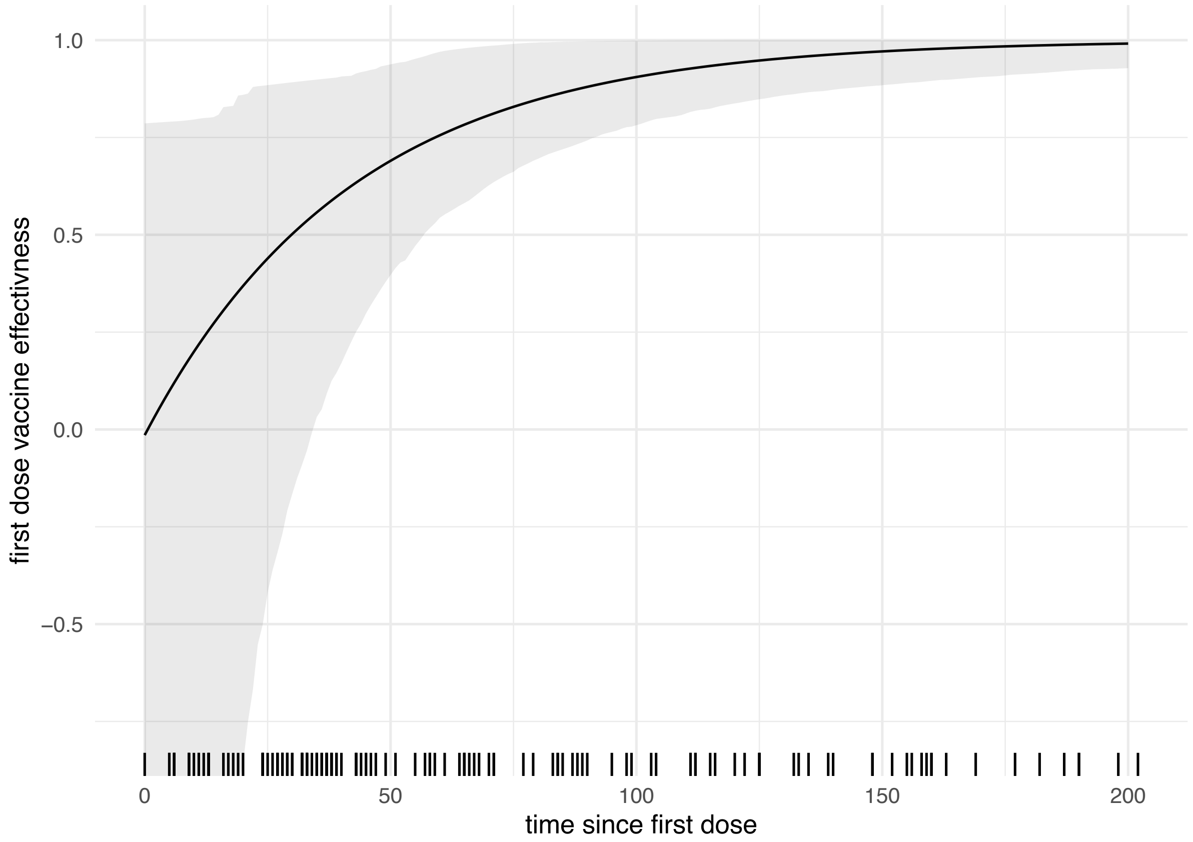

Figure S5. **Estimates (unadjusted) of one-dose protection over different assumed lag-times from test-negative design**. Grey envelopes represent 95% bootstrap confidence intervals. Horizontal lines on the x-axis represent days since each participant's first vaccine in the dataset. We did not fit adjusted models for all lags as many failed to converge due to limited data. Note that the x-axis is truncated at 200 days thought there are participants with a longer lag from their first dose.

Supplemental Tables

Table S1 **Vaccine coverage estimates from after the second vaccination round.** We conducted a coverage survey of all vaccine-eligible members of 403 households from 16 May 2022 through 30 June 2022 during a census of the entire camp population. We used systematic sampling, with a sampling interval of 30. Methods for ascertaining vaccination status were the same as those reported in Nesbitt et al, 2024.

	n	%
Households visited	403	
Vaccine-eligible individuals interviewed	1320	
**Vaccination Status***		
Unknown	5	0.3%
Unvaccinated	450	34.1%
One dose	217	16.4%
Two doses	648	49.1%
*Self-reported vaccination status. The first dose was confirmed by vaccination card among 552 of the 865 individuals reporting to have received at least one dose. Of those that reported having had two doses, 357 of them had a vaccination card.		

Table S2. **Comparison of characteristics and lab results from vaccine breakthrough and non-breakthrough confirmed cases**.

**Characteristic**	**Non-vaccinated** N = 10[1]	**Vaccinated** N = 11[1]	**p-value**[2]
Age (years)	19.0 (1.7)	22.6 (6.2)	0.05
Sex			1.00
Female	3 (30.0%)	4 (36.4%)	
Male	7 (70.0%)	7 (63.6%)	
Effective Doses			<0.0001
0	10 (100.0%)	0 (0.0%)	
1	0 (0.0%)	7 (63.6%)	
2	0 (0.0%)	4 (36.4%)	
Vaccination card available	-	6 (54.5%)	
ALT (IU/L)	21.7 (17.4, 1,140.0)	31.9 (15.5, 661.0)	1.00
Unknown	4	4	
Elevated ALT	2 (33.3%)	3 (42.9%)	1.00
Unknown	4	4	
Inpatient/Outpatient	2 (20.0%)	0 (0.0%)	0.21
Bilirubin (mg/dL)	6.8 (1.1, 12.0)	2.2 (1.0, 4.0)	0.34
Unknown	4	7	
PCR Positive			0.66
Positive	6 (60.0%)	8 (72.7%)	
Negative	4 (40.0%)	3 (27.3%)	
HEV CT Value	29.2 (4.3)	28.0 (5.0)	0.76
Unknown	5	4	
ELISA IgM Result			0.48
Positive	10 (100.0%)	9 (81.8%)	
Negative	0 (0.0%)	2 (18.2%)	

[1]Mean (SD); n (%); Median (Q1, Q3)

[2]Fisher's exact test; Wilcoxon rank sum test

Table S3. **Overview of enrolled test negative cases and their matched controls (bias-indicator study), including demographics, potential risk factors and vaccination status.**

**Characteristic**	**Test Negative Cases** N = 171[1]	**Matched Controls** N = 757[1]	**P-value**
Sex			0.17
Female	95 (55.6%)	450 (59.4%)	
Male	76 (44.4%)	307 (40.6%)	
Age (years)	25.5 (6.4)	24.8 (6.2)	0.64
Months in IDP Camp	83.6 (53.6, 97.0)	86.2 (67.5, 97.6)	0.0043
Size of Household	8.2 (2.7)	12.1 (54.6)	0.0003
Unknown	0	1	
Number of Children <5 in Household	2.1 (1.3)	2.3 (1.5)	0.067
Unknown	0	1	
Highest Education Level Achieved			<0.0001
None	71 (41.5%)	268 (35.5%)	
Primary school certificate	69 (40.4%)	305 (40.5%)	
Secondary School or Higher	31 (18.1%)	181 (24.0%)	
Unknown	0	3	
Drinking Water Source			1.00
Tap stand	170 (99.4%)	755 (99.9%)	
Surface water	0.0 (0.0%)	1 (0.1%)	
Borehole	1 (0.6%)	0 (0.0%)	
Unknown	0	1	
Defection Location			<0.0001
Shared Latrine	105 (61.4%)	729 (96.6%)	
Open Defecation	66 (38.6%)	26 (3.4%)	
Unknown	0	2	
Soap available in household for handwashing	116 (68.2%)	439 (58.7%)	0.051
Unknown	1	9	
Household member with AJS in last 2 months			
At least one	20 (11.7%)	47 (6.3%)	0.011
Unknown	0	5	
Share water source with someone with AJS	17 (10.0%)	44 (6.1%)	0.069
Unknown	1	36	
Effective Doses			<0.00001
0	42 (24.6%)	228 (30.3%)	
1	37 (21.6%)	125 (16.6%)	
2	88 (51.5%)	381 (50.7%)	
3	4 (2.3%)	18 (2.4%)	
Unknown	0	5	
Vaccination Card	70 (54.0%)	263 (52.0%)	

[1]n (%); Mean (SD); Median (Q1, Q3)

Table S4. **Estimates from bias indicator study using community controls and conditional logistic regression models**

	# Cases (n effective)[1]	# Controls (n effective)[2]	Cases Vaccinated (%)	Controls Vaccinated (%)	VE (unadjusted), 95% CI	VE (adjusted), 95% CI[3]
Two doses	130 (104)	464 (395)	88 (67.7%)	291 (62.7%)	-25.9 (-93.7, 18.2)	-30.9 (-102.5, 15.3)
At least one dose	171 (139)	752 (627)	129 (75.4%)	524 (69.7%)	-33.7 (-97.1, 9.3)	-42 (-110.3, 4.1)
One dose	79 (51)	164 (132)	37 (46.8%)	60 (36.6%)	-55.1 (-179.5, 13.9)	-78.3 (-231.6, 4.1)

[1]Effective controls are the number of controls that contribute to likelihood function in each model estimating VE. In the case of two-dose VE, only individuals with either zero or two doses of vaccine contribute information to the likelihood and those with one dose are effectively dropped from the model. In the case of one-dose VE, only those with one or zero doses contribute to the likelihood.

[2]While we aimed to enroll 6 controls per case, this was not always feasible, and some cases have slightly more or less than 6 controls.

[3]Adjusted for age and months living in camp as models with education did not converge.

Table S5. **Estimates of vaccine effectiveness (VE), lower and upper 95% confidence intervals, and AIC for models with restricted cubic splines for continuous variables**. Transformation column indicates the transformation variable (first in parenthesis) and the number of knots in the spline (second item in parenthesis after the comma).

Exposure	Transformation	VE	VE lower	VE upper	AIC
**Conditional Logistic Regression**					
Two doses	rcs(weeks_in_camp_std,3)	88.0	-158.6	99.4	25.67
Two doses	rcs(age_std,3)	94.0	-205.1	99.9	25.71
Two doses	rcs(age_std,3) + rcs(weeks_in_camp_std,3)	92.6	-236.1	99.8	25.81
Two doses	rcs(age_std,4)	92.1	-333.3	99.9	26.00
Two doses	none	84.0	-208.5	99.2	26.46
Two doses	rcs(age_std,4) + rcs(weeks_in_camp_std,3)	90.1	-349.1	99.8	27.01
Two doses	rcs(weeks_in_camp_std,4)	89.5	-188.0	99.6	27.65
Two doses	rcs(age_std,3) + rcs(weeks_in_camp_std,4)	93.7	-229.1	99.9	27.74
Two doses	rcs(age_std,4) + rcs(weeks_in_camp_std,4)	92.3	-295.3	99.9	28.60
**Unconditional Logistic Regression (Test Negative)**					
Two doses	none	89.4	56.4	98.0	68.58
Two doses	rcs(age_std,3)	88.0	50.3	97.7	68.78
Two doses	rcs(age_std,3) + rcs(weeks_in_camp_std,3)	91.4	60.4	98.7	68.86
Two doses	rcs(weeks_in_camp_std,3)	92.2	64.1	98.8	68.96
Two doses	rcs(age_std,4) + rcs(weeks_in_camp_std,3)	90.7	55.8	98.5	69.05
Two doses	rcs(age_std,4)	86.9	43.8	97.5	69.22
Two doses	rcs(age_std,4) + rcs(weeks_in_camp_std,4)	91.8	58.6	98.9	70.68
Two doses	rcs(age_std,3) + rcs(weeks_in_camp_std,4)	91.7	60.5	98.8	70.80
Two doses	rcs(weeks_in_camp_std,4)	92.0	63.3	98.8	70.82
One dose	rcs(age_std,4) + rcs(weeks_in_camp_std,3)	44.3	-113.6	87.3	77.24
One dose	rcs(age_std,4)	35.1	-139.7	83.9	77.42
One dose	rcs(age_std,4) + rcs(weeks_in_camp_std,4)	43.9	-115.9	87.2	79.13
One dose	rcs(age_std,3)	39.9	-106.7	84.0	81.46
One dose	rcs(weeks_in_camp_std,3)	42.7	-96.1	84.9	81.60
One dose	none	37.0	-108.3	82.4	81.80
One dose	rcs(age_std,3) + rcs(weeks_in_camp_std,3)	44.6	-94.7	85.9	81.94

Table S6. **Effectiveness estimates with alternative case definition where a case is any suspected case with ALT elevated ≥2.5 normal limit with at least one positive HEV test (ELISA IgM, RDT IgM, PCR or 4-fold IgG rise)**. Note that this is the same set of cases that are classified as positive by PCR alone.

	# Cases (n effective)[1]	# Controls (n effective)	Cases Vaccinated (%)	Controls Vaccinated (%)	VE (unadjusted), 95% CI	VE (adjusted), 95% CI[2]
Community-Matched Case Control Study						
Two doses	8 (5)	26 (21)	2 (25%)	13 (50%)	74.2 (-149.3, 97.3)	58.0 (-409.8, 96.5)
At least one dose	14 (10)	82 (58)	8 (57.1%)	62 (75.6%)	59.8 (-49.7, 89.2)	22.4 (-223.6, 81.4)
One dose	12 (9)	37 (35)	6 (50%)	19 (51.4%)	43.2 (-143.5, 86.8)	19.9 (-296.1, 83.8)
Test-Negative Design (Facility Controls)						
Two doses	8 (8)	130 (130)	2 (25%)	88 (67.7%)	84.1 (27.6, 97.7)	91.9 (52.1, 99.1)
At least one dose	14 (14)	171 (171)	8 (57.1%)	129 (75.4%)	56.6 (-38.5, 85.7)	63.0 (-29.6, 89.3)
One dose	12 (12)	79 (79)	6 (50%)	37 (46.8%)	-13.5 (-292.3, 67.1)	-1.2 (-271.3, 72.4)

[1]Effective controls are the number of controls that contribute to the estimates of VE. In the case of two-dose VE, only those with either zero or two doses of vaccine contribute information. In the case of one-dose VE, only those with one or zero doses contribute.

[2]Adjusted for age, sex and months living in camp
